# Impact of Traditional Behavior of Customers, Employees, and Social Enterprises on the Fear of Change and Resistance to Innovation

**DOI:** 10.3389/fpsyg.2022.923094

**Published:** 2022-07-26

**Authors:** Xiaoyan Liu, Fei Wang, CheeHoo Wong

**Affiliations:** ^1^International Education College, Hebei Finance University, Baoding, China; ^2^School of Foreign Languages for International Business, Hebei Finance University, Baoding, China; ^3^Faculty of Business and Communications, INTI International University, Nilai, Malaysia

**Keywords:** social enterprises, resistance to innovation, customers' traditional behavior, employees' traditional behavior, social enterprises' traditional behavior, China

## Abstract

Innovation adoption is the necessary element for the success of any organization around the globe, and this phenomenon needs a foremost solution. The current study examines this area and explores the impact of customers, employees, and social enterprises' traditional behavior on the resistance to innovation in social enterprises in China. The current article also investigates the mediating role of fear for change among customers, employees, and social enterprises' traditional behavior and resistance to innovation in social enterprises in China. This article has followed the primary data gathering methods and adopted the questionnaires for this purpose. The employees and customers of social enterprises are the respondents and ~11,000 population in the study. According to Krejcie & Morgan, the sample size criteria is around 370. Thus, the researchers' have forwarded around 615 surveys and received only 357 after a few weeks. The present research has also applied the SPSS-AMOS to analyze the association among variables and test the hypotheses. The results revealed that the traditional behavior of customers, employees, and social enterprises has a significant and positive linkage with resistance to innovation in social enterprises in China. The findings also exposed that the fear of change also significantly mediates among customers, employees, and social enterprises' traditional behavior and resistance to innovation in social enterprises in China. This study helps the regulators establish policies related to innovation adoption by changing traditional behavior to advance the behavior of customers, employees, and social enterprises.

## Introduction

The competition between the firms in the world has accelerated at a rapid pace. Firms all around the globe are investing their maximum efforts to secure a competitive advantage with the view to winning the competition. One of the factors which help the organization secure a competitive advantage is the proper research and development. The ultimate product of research and development is innovation. Innovation is all about change. Innovation leads to competitive advantage (Udriyah et al., 2019; Chien et al., 2021b). Innovation is all about change. The concept of acceptance of change is rated very high in all the forums (Chien et al., 2021c). Human psychology narrates that acceptance of change is a difficult process. From the organizational point of view, the resistance to innovation is faced by customers, employees, social enterprises, and internal as well as external stakeholders. One of the core factors that stands behind the innovation resistance is the individual's psyche which usually forces the individuals to not accept the change. Another factor is that employees or customers used to avoid breaking their comfort zone. The common factors which caused to resist the innovation are (1) personality, (2) attitudes, (3) value orientation, (4) previous innovative experience, (5) perception, (6) motivation, and (7) psychological factors (Dibrov, 2015; Zhang, 2019). Literature proposed that the organizations which introduced the innovation before its preparation usually fail to innovation acceptance (Rodríguez Sánchez et al., 2019; Chien et al., 2021a). Several factors play a vital role in the betterment of the country's economy. Some work at large while others at a small scale. The sector which has its impact from top to bottom of a mediocre section of the society is small and medium enterprise. Social enterprises have a short reaction time due to their high adaptability and flexibility. Due to fewer resources and investments, social enterprises usually avoid innovation. This is one of the prime factors that the present study selected the social enterprises.

China is one of the production giants in the world. The government of China with the view to support its people as well as its economy facilitate all level of enterprises at the maximum level. Social enterprises are considered as one of the tools to financially support the bottom level of society and enhance their standard of living (Pandey, 2020). From the world's point of view, social enterprises play a significant role in the majority of economies, particularly in developing countries. Social enterprises make for the vast majority of businesses globally and are critical contributors to employment creation and global economic development. They account for around 90% of enterprises and more than 50% of global employment. In emerging economies, formal social enterprises can account for up to 40% of national income (GDP). Although social enterprises are a significant player in the Chinese economy they are also facing significant issues in terms of innovation acceptance. The social enterprises are owned and run by the lover and mediocre level of the society. However, social enterprises have a lack of skilled employees along with a lack of financial support, which brings fear for them to accept any kind of change in the form of innovation (Supriyadi et al., 2018). This situation exposed the resistance to innovation situation among the social enterprises in China and needs to resolve these issues. The current study examines this area using customers' employees' and enterprises' traditional behavior related to innovation adoption.

The present study will address some gaps does exist in the literature like (1) being one of the important and liked concept behavior and innovation although researched although but still not reached its peak, (2) Rodríguez Sánchez et al. (2019), investigated the customers resistance toward the innovation in the tourism industry whereas the present study will test the association of resistance to innovation with customer, employee and social enterprises behavior in Chinese social enterprises, (3) Sun (2021), worked customer churn and resistance whereas the present study will test innovation resistance with different behaviors along with the addition of mediation factor like fear of change in China, (4) Dibrov (2015), worked on the factors of overcoming innovation resistance whereas the present study will test the association of resistance to innovation with customer, employee and social enterprises behavior in Chinese social enterprises with an updated data set, (5) the model is not tested before in China that why the present study will check the model in China perspective with new data set, (6) Lipych et al. (2018), worked on the employees innovation behavior whereas the present study will work with mediation effect in China. The significance of the study are (1) will highlight the importance of performance in Chinese social enterprises, (2) help the professional to revamp their policies for acceptance of innovation as well as the betterment of the performance in social enterprises of China, and (3) will help the researchers to identify the reasons stands behind the resistance to innovation and its importance for industries prevailing in the country economic.

The study structure is divided into five phases. The first phase will present the introduction. In the second phase of the study, the pieces of evidence regarding customer traditional behavior, employee traditional behavior, social enterprises traditional behavior, fear of change, and resistance to innovation will be discussed in the light of past literature. The third phase of the study will shine the spotlight on the methodology employed for the collection of data regarding customer traditional behavior, employee traditional behavior, social enterprises traditional behavior, fear of change, and resistance to innovation and its validity will be analyzed. In the fourth phase, the results of the study will be compared with the pieces of evidence reviewed from the literature. In the last phase, the study implications along with the conclusion and future recommendations will be presented which will conclude the paper.

## Literature Review

The world is fond of behaviors and the changing perspectives of customers in the world usually force the tradition itself. Ultimately the traditional behavior belongs to the people of China and the rising trend of tradition as well as innovation and technology helps to move toward advancement. Thus, Suhud et al. (2020) analyzed the consumer behavior in the traditional markets and the measurement of the resistance to innovation. Chinese people are usually maintaining their focus on innovation but the traditional behaviors in some backward areas are resisting to do so. In this context, the digital views of the innovation have not only helped the people to move toward technology but also helped in adapting to the innovation. Furthermore, Ooi and Husted (2021) assessed the capabilities and behaviors of consumers in traditional means and their impact on product innovation. The various facilities that are usually provided in the changing world helped in changing the traditional behavior of customers that dominates over resistance toward innovation. Although the traditional behavior is reluctant to be changed in China, the features, availability, and prices of products highly convinced the customers. Umbrello (2018) expressed the drivers of consumers' behaviors toward smart products, which are resisted due to the innovation. While the customer perspectives are changed, the resistance to innovation is significantly eliminated. This helped the Chinese people to fight the resisted elements which are the main barrier to innovation and moving toward technology.

**H1:** Customers' traditional behavior significantly impacts the resistance to innovation.

Organizations frequently adapt to the changes in their structures as well as in their operations and this is due to meet the challenges in the changing world. Therefore, China has highly and at peak level changed its technologies and moved toward significant steps to counter the threats. In this context, Ababneh (2021) investigated the traditional behaviors of employees in an organization that are the main resistance toward innovation. In the industries of China, the traditional behavior of employees has been considered the main factor for this applicability of change. Although, the change is sometimes uncertain and unable to be imposed in an organization better technology supports the verdict of countering challenges. Additionally, Li and Tian (2016) assessed the influence of employee behaviors in a workplace that is evaluated from different aspects as resistance to innovation. Traditionally, the products in the organization change and the change requires innovation therefore, the employee behavior is necessary to be changed. When the employees are skillful and retain motivation to move toward technology, then the pending elements could easily be motivated by resistance. Moreover, Stryja and Satzger (2019) examined the fairness and effectiveness of decisions due to the traditional employee behavior and its resistance toward innovation. The establishment of online stores in China is a better example for the people and employees who fear change and the change always require mutual support. Employee traditional behavior could not be singly moved and instantly the mutual effort is an advantage in eradicating the resistance to innovation.

**H2:** Employee traditional behavior significantly impacts the resistance to innovation.

The developing world has induced numerous entrepreneurship programs and efforts that motivated small business people to adapt to changes. In China, the chain of social enterprise's traditional behaviors has been so much changed that people feel realistic to approach them. Therefore, Nguyen (2021) viewed the networks and support of social traditional behaviors that drop significant influence on the resistance to innovation. These enterprises are segregated into various aspects whether these enterprises belong to the local area or the innovative area. Therefore, the context of traditional behavior could easily be elaborated in the eyes of people who are unable to meet the standards of innovation. Thus, Kulis et al. (2018) examined the relationship between gender roles with the relevance of traditional behaviors over the enterprises and resistance to innovation. The standards of innovation only require exposure of ideas and the facilities that upgrade the image of people. Ultimately, the social enterprises that are acquainted with the traditional behavior are usually the main resistance to innovation. Additionally, Park et al. (2018) investigated the experience and acceptance of innovation which is resisted by some perceived attributes of traditional social aspects. This resistance requires up-gradation in traditional behaviors through which the resistance to innovation could easily be eradicated. The social enterprise's traditional behavior is termed as the biggest barrier and resistance toward the innovative environment in some cities of China. Many backward people and social enterprises are gradually but increasingly trying to move toward innovation after significant steps taken by the government of China.

**H3:** Social enterprises' traditional behavior significantly impacts the resistance to innovation.

Manifests of countries are important when the struggle of countries reaches some point because the neighboring countries always require the manifesto. Therefore, in the countries that adopt prominent steps and policies following the changing world, then technology could play its role. In this context, Sarrina Li and Huang (2020) discussed the relationship between behavioral intentions, information processing, and fear appeals that insert a role in traditional behavior and innovation. Technology has somehow changed the perspectives of business individuals as well as the customers due to the involvement of innovation. There is the element of fear of change in people of china that are considered the main element resisting innovation. Similarly, Bottaccioli et al. (2019) analyzed the traditional consumer. The role of fear of change has certainly inserted a significant role in the customers' traditional behaviors and among the people that are resisting innovation. This inducement of fear of change which changed the perspectives of people also induced the fear of doing business. Finally, Raajpoot and Sharma (2021) enumerated the functioning of innovative culture that strives toward the success of new products and services by breaking barriers of resistance. The customers' perspective could only be changed when the element of fear of change is properly taken into consideration and proper facilitation is given to the customers about the innovation. The mediating impact of fear of change is indicating the changing traditional behavior perspective of customers that resist innovation.

**H4:** Fear of change significantly and positively mediates the relationship between customers' traditional behavior and resistance to innovation.

Business always requires risk and the companies and organization that has moved toward the innovation have prominently achieved more targets. Therefore, the organizations in China have achieved more targets as compared to the other world. In this context: Oplatka and Iglan (2020), enumerated the emotions of fears and their implications on the resistance to innovation and behaviors. It is just due to the significant and consistent adaptation of the innovative approach. The fear of change in China is also considered a dominant element among the employees whose behavior certainly changes due to changes in tradition. Moreover, Oyetunde et al. (2022) assessed the relationships between traditional and non-traditional employment and its contribution to the resistance to innovation. However, there is also the tradition of employees in an organization about the workings and the ways of workings. Technology has facilitated the organizations to achieve their objectives more precisely than the organizations that had not acquainted with the technological advancement. Consequently, Mani and Chouk (2019) examined the concerns of privacy that prevail in the organizations, and employee traditional behavior poses some resistance. In China, the traditional behavior of employees is the main hindrance to the movement to innovation. This hindrance is just due to the lack of involvement in technological measures and these measures could be eliminated by the mediating role of fear of change. This element of fear of change is a feasible aspect to enumerate the relationship and solutions between employee traditional behavior and resistance to innovation.

**H5:** Fear of change significantly and positively mediates the relationship between employees' traditional behavior and resistance to innovation.

Social culture and tradition in China convey more importance in the countries where the people are not ready to acquaint themselves with the change. This is just due to the lack of facilities and guidance provided in the remote areas of China. Thus, Pittman et al. (2021) analyzed the changing attitudes due to the effects of information and the fear of the consumers resisting innovation. The fear of change takes place in the social enterprises which have not used innovativeness and technology. The prevalence of fear of change inserts clear destruction to the innovation and the countries could not move forward with technology. Moreover, Sone (2018) examined the factors of social enterprises where traditional behavior is necessary to emphasize the fear of change along with innovation. While viewing the elements of fear of change, the progressive approach of social online stores has imported an idea from the traditional behaviors. This importation has not only convinced the social enterprise's traditional behavior to stop resistance toward innovation but also forced them to take necessary steps. Finally, Zhang (2019) assessed the views of the relationship between enemies and innovation where the technologies are resisted due to social enterprise behaviors. These necessary steps accumulate important innovation and technological measures for forming feasible and strong manifests among the social enterprises. The people are just establishing the element of fear of change because of the lack of information about the importance of innovation. Therefore, fear of change not only helps to take progressive steps but also helps to prepare in countering threats.

**H6:** Fear of change significantly and positively mediates the relationship between social enterprises' traditional behavior and resistance to innovation.

## Methods and Material

**Figure 1:**
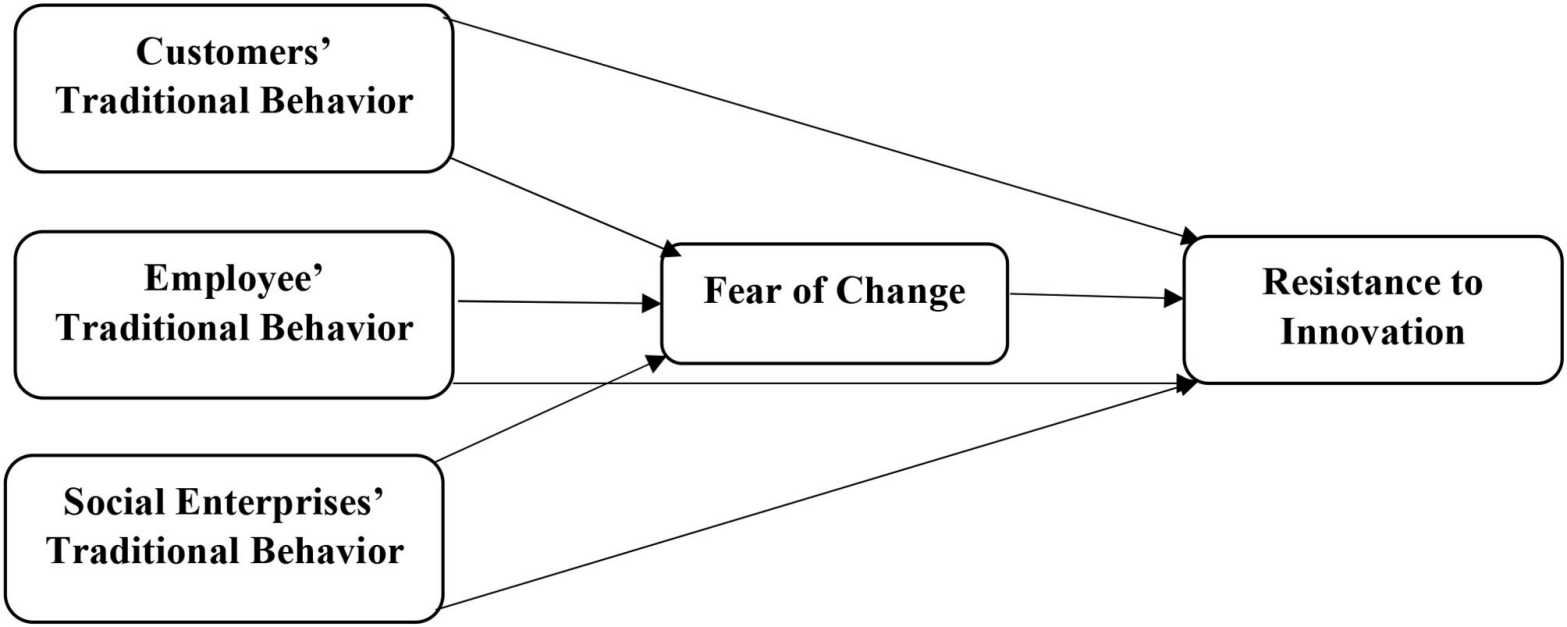
Theoretical model.

The article explores the impact of customers, employees, and social enterprises' traditional behavior on the resistance to innovation and investigates the mediating role of fear for change among customers, employees, and social enterprises' traditional behavior and resistance to innovation in social enterprises in China. This study follows the primary data gathering methods and adopted the questionnaires for this purpose. The current article has taken three predictors such as customers' traditional behavior, employees' traditional behavior, and social enterprises' traditional behavior. In addition, the fear of change (FC) is taken as mediating construct, and resistance to innovation has been taken as a dependent variable. The current study has developed the theoretical framework using behavioral theory. The behavioral theory describes human behavior to adopt or reject a particular thing using previous experience. The current study has also examined the customers' employees' and social enterprises' behavior to resistance to innovation due to fear of change that they experience in the past. Thus, based on behavioral theory, the present study has developed the theoretical framework shown in Figure 1.

The current article has taken customers' traditional behavior (CTB) as the predictor with four items taken from Li (2018). These four items are mentioned in Table 1. In addition, the current article has taken employees' traditional behavior (ETB) with four items and taken from Farrukh et al. (2020). These four items are mentioned in Table 1. Moreover, the current article has taken social enterprises' traditional behavior (SETB) with four items extracted from Li et al. (2020). These four items are mentioned in Table 1. Additionally, the current article has taken the fear of change (FC) as mediating construct with three items taken from George et al. (2020). These three items are mentioned in Table 1. Finally, the current article has taken resistance to innovation (RTI) as a dependent variable that has eight items extracted from Hosseini et al. (2016). These eight items are mentioned in Table 1.

**Table 1:** Measurements of the variables.

**Items**	**Statements**	**Sources**
**Consumer traditional behavior**		
CTB1	“I no need to get up-to-date information related to the product.”	(Li, 2018)
CTB2	“I find the low price product not to focus on a high-quality product.”	
CTB3	“I spend less by using the old product.”	
CTB4	“I am not determined to switch to quality products.”	
**Employees' traditional behavior**		
ETB1	“I am happy to work with existing conditions.”	(Farrukh et al., 2020)
ETB2	“I am always afraid when new work is given to me.”	
ETB3	“I am not willing to work with changing conditions.”	
ETB4	“I am not able to adopt new technology in my work.”	
**Social enterprises traditional behavior**		
SETB1	“My organization is not willing to adopt changes in the working process.”	(Li et al., 2020)
SETB2	“My organizational follows the traditional way of working.”	
SETB3	“My organization provides a traditional working environment.”	
SETB4	“My organization is unwilling to provide us workshops, training or any other facility to improve our work.”	
**Fear of change**		
FC1	“I am worried when a new task is allowed to me by the authorities.”	(George et al., 2020)
FC2	“My organizational also fears to change in the process due to higher cost.”	
FC3	“Stakeholders also worried about adopting new ideas because of lack of knowledge and finance.”	
**Resistance to innovation**		
RTI1	“I will wait to adopt new technology until it proves beneficial.”	(Hosseini et al., 2016)
RTI2	“I need to clarify some queries and justify the reasons to adopt new technology.”	
RTI3	“I am waiting for the right time and required capability to adopt new technology.”	
RTI4	“I fear wasting my time using new technology.”	
RTI5	“I need to get a solution for some of my complaints and objections before I adopt new technology.”	
RTI6	“I fear certain changes in the organization may impose on me.”	
RTI7	“Innovation is not for me.”	
RTI8	“It is unlikely that I will adopt innovation in the near future.”	

**Figure 2:**
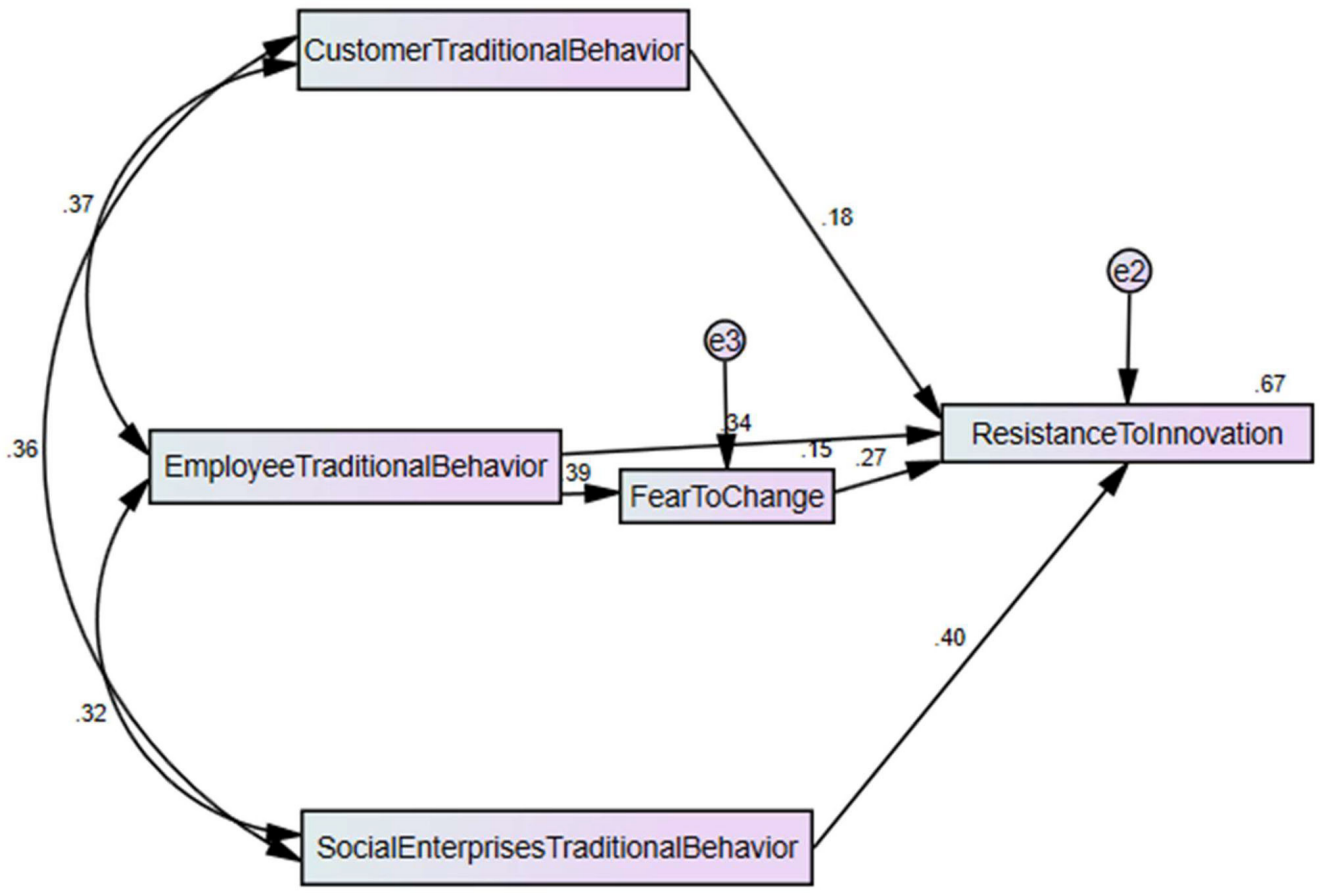
Structural model assessment.

These questionnaires were sent to the selected respondents using mail and personal visits to the social enterprises in China. The customers and employees of social enterprises are the respondents and ~11,000 population in the study. According to the Krejcie & Morgan sample size criteria, the sample size is around 370. The part of the questionnaire related to the customers' traditional behavior was for the customers, while the remaining part of the questionnaire was related to the employees. These respondents are chosen using simple random sampling. The researchers have forwarded around 615 surveys and received only 357 after a few weeks. These surveys have a 58.05% response rate. The present research has also applied the SPSS-AMOS to analyze the association among variables and test the hypotheses. This is an effective tool that provides effective results even though the authors have used complex models or large sample sizes (Purwanto et al., 2021). Figure 2 shows the association among variables and indicated positive associations among the variables. In addition, Figure 2 also shows the positive mediation impact of fear to change among predictors and dependent variable.

## Research Findings

The study has shown the descriptive statistics that exposed 251 of the respondents are male while 106 of the respondents are female. In addition, descriptive statistics also revealed that 102 of the respondents have graduation qualifications while 221 of the respondents have master's qualifications, and 34 of the respondents have other qualifications than graduation and master's. Finally, descriptive statistics also revealed that 187 of the respondents have 0–5 years of experience, while 221 of the respondents have 6–10 years of experience, and 34 of the respondents have above 10 years of experience (Table 2).

**Table 2:** Descriptive statistics.

**Gender**			
Male	Female	Total	
251	106	357	
**Qualifications**			
Graduation	Masters	Others	Total
102	221	34	357
**Experiences**			
0–5 Years	6–10 Years	Above 10 Years	Total
187	114	56	357

This article has applied the average variance extracted (AVE) to examine the convergent validity, and statistics revealed that the AVE figures are more than 0.70. These outcomes exposed the convergent validity as valid. This article has also applied the composite reliability (CR) to examine the reliability, and statistics revealed that the CR figures are more than 0.70. These outcomes exposed significant reliability. This study applies factor loadings to examine the content validity, and statistics revealed that the figure values are more than 0.50. These outcomes exposed the content validity as valid. Table 3 shows all of the stated figures.

**Table 3:** Convergent validity.

**Constructs**	**Items**	**Loadings**	**CR**	**AVE**
Customer traditional behavior	CTB4	0.746	0.927	0.763
	CTB3	0.961		
	CTB2	1.001		
	CTB1	0.755		
Employee traditional behavior	ETB4	0.921	0.946	0.814
	ETB3	0.965		
	ETB2	0.833		
	ETB1	0.884		
	SETB4	0.975	0.957	0.847
Social enterprises traditional behavior	SETB3	0.991		
	SETB2	0.831		
	SETB1	0.875		
Fear to change	FC3	0.575	0.749	0.503
	FC2	0.741		
	FC1	0.794		
Resistance to innovation	RTI8	0.777	0.888	0.523
	RTI7	0.791		
	RTI6	0.675		
	RTI5	0.653		
	RTI4	0.636		
	RTI3	0.666		
	RTI2	0.741		
	RTI1	0.700		

This study has applied the Fornell Larcker criteria to examine the discriminant validity, and statistics revealed that the first value in the column is larger than the other values in the column. These outcomes exposed the discriminant validity as valid. Table 4 shows all of the stated figures.

**Table 4:** Discriminant validity.

	**RTI**	**CTB**	**ETB**	**SETB**	**FC**
RTI	0.707				
CTB	0.511	0.874			
ETB	0.703	0.349	0.902		
SETB	0.663	0.355	0.313	0.920	
FC	0.689	0.377	0.453	0.444	0.709

The current study has checked the good model fitness using the Tucker–Lewis index (TLI), and the figure is >0.90, and the exposed model is a good fit. In addition, the comparative fit index (CFI) is also used to check the model's good fitness, and the figure is larger than 0.90 and indicates the model is a good fit. Finally, the results also checked the model good fitness using root mean square error of approximation (RMSEA) and indicated the value is 0.07 while the acceptable and exposed model is acceptable. Table 5 shows these figures.

**Table 5:** Threshold of goodness-of-fit indices.

**Selected indices**	**Result**	**Acceptable level of fit**
TLI	0.965	TLI > 0.90
CFI	0.973	CFI > 0.90
RMSEA	0.070	RMSEA <0.05 good; 0.05–0.10 acceptable

**Figure 3:**
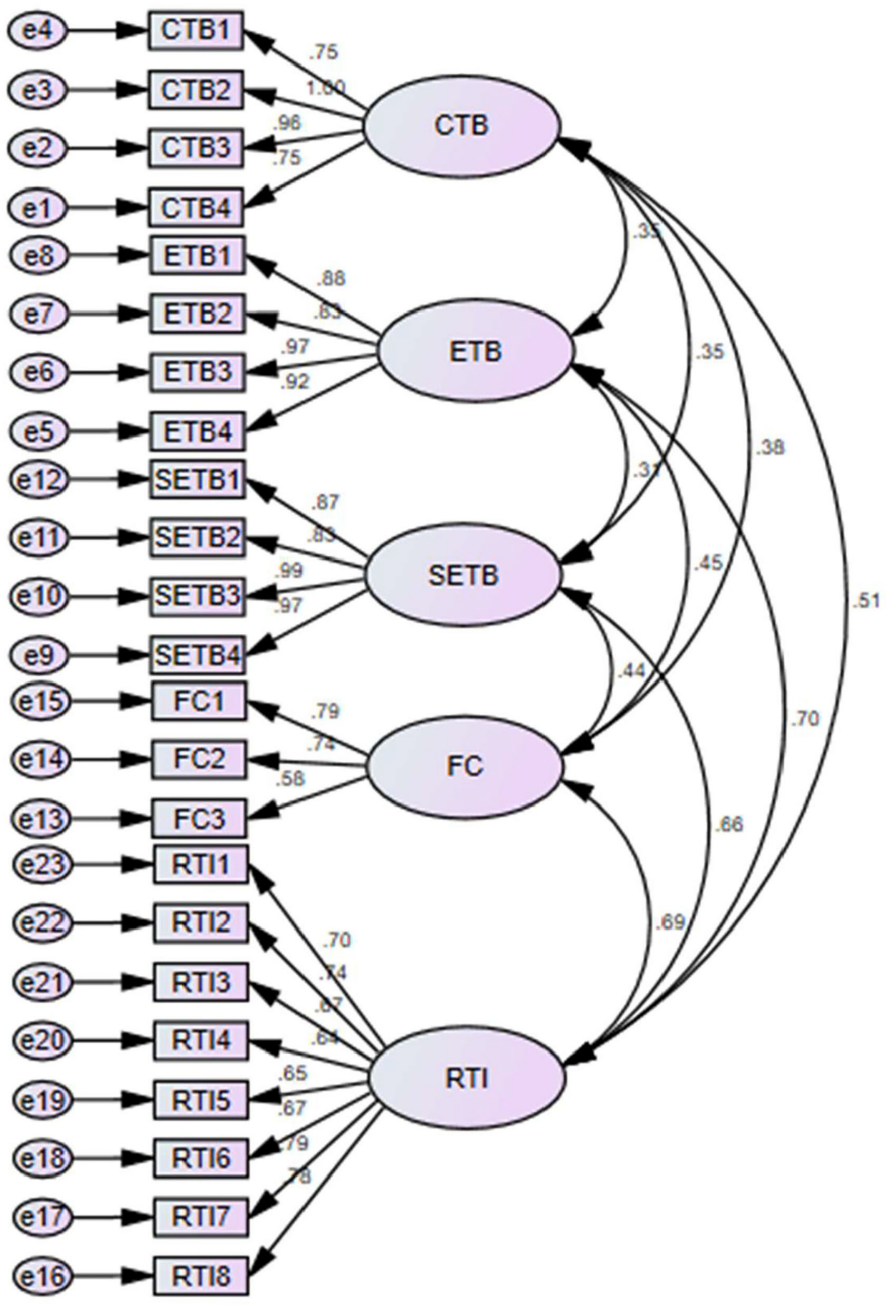
Measurement model assessment.

The results of the direct path revealed that customers, employees, and social enterprises' traditional behavior have a significant and positive linkage with resistance to innovation in China and accept H1, H2, and H3. Table 6 given below shows all of the stated figures. Figure 3 provided the factor loadings and results indicated that the values are higher than 0.50 and exposed valid content validity.

**Table 6:** Direct path analyses.

**Relationships**			**Std. Beta**	**Beta**	**S.E**.	**C.R**.	***p***
Fear to change	< –	Employee traditional behavior	0.386	0.339	0.043	7.895	^***^
Resistance to innovation	< –	Customer traditional behavior	0.181	0.133	0.025	5.333	^***^
Resistance to innovation	< –	Social enterprises traditional behavior	0.399	0.282	0.023	12.049	^***^
Resistance to innovation	< –	Employee traditional behavior	0.342	0.286	0.030	9.571	^***^
Resistance to innovation	< –	Fear to change	0.267	0.255	0.031	8.144	^***^

^***^, ^**^, and ^*^ represent significant level at 1%, 5%, and 10%, respectively.

The findings of mediation analysis also exposed that the fear of change also significantly mediates among customers, employees, and social enterprises' traditional behavior and resistance to innovation in China and accept H4, H5, and H6. Table 7 given below shows all of the stated figures.

**Table 7:** Mediation analysis.

	**CTB**		**ETB**		**SETB**	
	**Beta**	***p***	**Beta**	***p***	**Beta**	***p***
Total effects	0.354	0.001	0.543	0.000	0.533	0.000
Direct effects	0.331	0.012	0.422	0.000	0.129	0.012
Indirect effects	0.292	0.023	0.047	0.003	0.422	0.003

## Discussions

The results exposed that the customers' traditional behavior has a positive impact on the resistance to innovation in social enterprises in China. Because of the customers' traditional nature are unwilling to adopt changes in the product, which is the reason for their positive link with the resistance to innovation. This results in line with Rodriguez Sanchez et al. (2020) also explore the association between customers' behavior role in innovation adoption and conclude that the traditional nature of customers is always reluctant to adopt innovation because they are unwilling to adopt changes in the products. In addition, this outcome is also similar to Roy et al. (2018), who also examine the impact of customers' behavior toward innovation adoption, and the traditional nature of the customers are unwilling to adopt the innovation because they are not used to and able to use new products that adopt innovation. Moreover, this outcome is also similar to the study by Chouk and Mani (2019), who also analyzed the customers' behavior toward innovation adoption and exposed that the customers who have traditional nature of behavior are always reluctant to adopt innovation because they are not used to and also not able to use these types of product.

The findings exposed that the employees' traditional behavior positively influences the resistance to innovation in social enterprises. Especially the old employees who are not used to technology and fear adopting it due to their unwillingness due to their traditional behavior that is the reason employees' traditional behavior has a positive impact on the resistance to innovation. This outcome is in line with Heidenreich and Talke (2020) also investigated the role of employees' nature and adoption of innovation and revealed that the traditional nature of employees is always unwilling to adopt the innovation because the adoption of innovation needs extra efforts to understand the work and they are unwilling to put extra time and happy with the existing ways of operations. In addition, this result is also similar to Bäckström and Bengtsson (2019) also examined the impact of employees willing to adopt innovation and revealed that the employees who are not motivated and the employees who are not updated according to the situation and have traditional mind always reluctant to adopt the innovation in the processes.

The results indicated that the social enterprises' traditional behavior has a positive effect on the resistance to innovation. Because the traditional nature of enterprises is unwilling to adopt changes in the processes due to high cost and extra efforts, that is the reason for their positive link with the resistance to innovation. This result is in line with the study by Richter (2019), who also explored the association between social enterprises' behavior role in innovation adoption and concluded that the traditional nature of social enterprises is always reluctant to adopt innovation because they are unwilling to adopt changes in the processes due to many reasons such as extra cost, lack of finance, extra efforts and trained workforce. In addition, this outcome is also similar to Joachim et al. (2018) also examine the impact of social enterprises' behavior toward innovation adoption, and the traditional nature of social enterprises are unwilling to adopt the innovation because they have lack of sources to adopt innovation in the business processes. Moreover, this outcome is also similar to the Apostolopoulos et al. (2019) study, who also analyzed the social enterprises' behavior toward innovation adoption and exposed that the social enterprises who have traditional nature of behavior are always reluctant to adopt innovation because they have lack of sources to adopt innovation in the business processes.

The results also investigated that the fear of change significantly and positively mediates customers' traditional behavior and resistance to innovation in the social enterprises in China. The customers who have traditional nature always fear changing nature, which forces them to resist the adoption of innovation in the products. This outcome is matched with Kaur et al. (2020) also investigated the customers' nature and adoption of innovation and concluded that the traditional nature of customers always has a fear of change nature moves them toward resistance to innovation. In addition, the findings also exposed that the fear of change significantly mediates employees' traditional behavior and resistance to innovation. Because the employees' traditional nature injects fear related to adopting the changes that force them toward resistance to innovation. This result is similar to Jung et al. (2020) also investigated the employees' behavior toward innovation adoption and revealed that the old nature of the employees has a high level of fear to change that is the research of resistance to innovation adoption. Moreover, the results also investigated that the fear of change significantly and positively mediates social enterprises' traditional behavior and resistance to innovation. The social enterprises that have traditional nature always fear changing nature due to high cost and lack of resources that force them to resist the adoption of innovation in the business processes. This outcome matches the study of Cohen et al. (2019), who also investigated the social enterprises' nature and adoption of innovation and concluded that the traditional nature of social enterprises always has a fear of change nature that moves them toward resistance to innovation.

## Implications and Limitations

The present study has some theoretical contributions along with the practical implication of the ongoing study. The present study contributes to the literature on customers' traditional behavior and resistance to innovation using the social enterprise context. In addition, the present article also contributes to the existing literature on employees' traditional behavior and resistance to innovation by using Chinese social enterprises. Moreover, the current article also contributes by providing the literature related to the social enterprises' traditional nature and resistance to innovation. In addition, it is one of the first attempts to adopt three predictors, such as customers' traditional behavior, employees' traditional behavior, and social enterprises' traditional behavior, to predict resistance to innovation. Fear of change is used as mediating variable and is also a significant contribution to the existing literature. The current study guides the policy implementation authorities to implement the policies that motivate the stakeholders to adopt the innovation. In contrast, the present study provides help to upcoming researchers to examine this area in the future. In addition, this study helps the regulators establish policies related to innovation adoption by changing traditional behavior to advance the behavior of customers, employees, and social enterprises. The article also suggested that the authorities should provide training sessions for the customers and employees that can generate extra resources to initiate change behavior and reduce their resistance to innovation. The article also suggested that social enterprises should provide extra incentives to cope with innovation and reduce the fear of change among customers and employees.

The present study also has some limitations that help the future studies for further investigations. The current research has used three predictors: customers' traditional behavior, employees' traditional behavior, and social enterprises' traditional behavior and ignores other factors and suggests that future studies must add these factors to predict resistance to innovation. In addition, the present study ignored the moderating role in the framework and recommended that future studies should incorporate this aspect in their analysis. Finally, the current study examined the social enterprises, ignored other industries, and recommended that future studies add more industries to expand their scope.

## Data Availability Statement

The original contributions presented in the study are included in the article/supplementary material, further inquiries can be directed to the corresponding authors.

## Author Contributions

This idea was given by FW. FW and CW analyzed the data and wrote the complete paper. While XL read and approved the final version. All authors contributed to the article and approved the submitted version.

## Conflict of Interest

The authors declare that the research was conducted in the absence of any commercial or financial relationships that could be construed as a potential conflict of interest.

## Publisher's Note

All claims expressed in this article are solely those of the authors and do not necessarily represent those of their affiliated organizations, or those of the publisher, the editors and the reviewers. Any product that may be evaluated in this article, or claim that may be made by its manufacturer, is not guaranteed or endorsed by the publisher.
